# Seroconversion for Infectious Pathogens among UK Military Personnel Deployed to Afghanistan, 2008–2011

**DOI:** 10.3201/eid2012.131830

**Published:** 2014-12

**Authors:** Edmund N.C. Newman, Penelope Johnstone, Hannah Bridge, Deborah Wright, Lisa Jameson, Andrew Bosworth, Rebecca Hatch, Jenny Hayward-Karlsson, Jane Osborne, Mark S. Bailey, Andrew Green, David Ross, Tim Brooks, Roger Hewson

**Affiliations:** Public Health England, Porton Down, UK (E.N.C. Newman, D. Wright, L. Jameson, A. Bosworth, J. Hayward-Karlsson, J. Osborne, T. Brooks, R. Hewson);; Queen Alexandra Hospital, Portsmouth, UK (P. Johnstone, H. Bridge);; BMS Training Defence School of Healthcare Education, Birmingham, UK (R. Hatch);; Royal Centre for Defence Medicine, Birmingham (M.S. Bailey, A. Green);; Army Health Unit, Camberley, UK (D. Ross);; National Institute for Health Research, Health Protection Research Unit in Emerging and Zoonotic Infections, Liverpool, UK (T. Brooks, R. Hewson);; London School Hygiene and Tropical Medicine, London, UK (R. Hewson)

**Keywords:** Afghanistan, serosurveillance, biosurveillance, hantavirus, Crimean-Congo hemorrhagic fever virus, CCHFV, Rickettsia, rickettsiae, Q fever, Coxiella burnetii, sandfly fever virus, undifferentiated fevers, United Kingdom, military, deployment, bacteria, viruses, seroconversion

## Abstract

Military personnel are at high risk of contracting vector-borne and zoonotic infections, particularly during overseas deployments, when they may be exposed to endemic or emerging infections not prevalent in their native countries. We conducted seroprevalence testing of 467 UK military personnel deployed to Helmand Province, Afghanistan, during 2008–2011 and found that up to 3.1% showed seroconversion for infection with *Rickettsia* spp., *Coxiella burnetii*, sandfly fever virus, or hantavirus; none showed seroconversion for infection with Crimean-Congo hemorrhagic fever virus. Most seroconversions occurred in personnel who did not report illness, except for those with hantavirus (70% symptomatic). These results indicate that many exposures to infectious pathogens, and potentially infections resulting from those exposures, may go unreported. Our findings reinforce the need for continued surveillance of military personnel and for education of health care providers to help recognize and prevent illnesses and transmission of pathogens during and after overseas deployments.

## Abstract 2

Many exposures, and potentially infections, are unreported.

Military personnel represent a population that travel to and work in environments where they are exposed to endemic or emerging infections that are not prevalent in their native country. Groups of military personnel on international deployments thus form a useful naive sentinel group for infectious diseases; these groups may serve as indicators of the infectious disease agents to which the local populations are exposed and of the diseases that may be encountered by other visitors traveling to the area (e.g., tourists, as well as staff of nongovernmental organizations, aid organizations, and other government agencies). Study of infectious disease exposures and illnesses among military personnel may provide insight into the epidemiology of emerging and reemerging infections and highlight what pathogens could be imported back to the home nations of visitors to these areas.

The recognition that military personnel may act as disease sentinels is not a new concept. Infectious diseases have beleaguered international military operations for centuries, with historic campaigns facing substantial loss of life after soldiers succumbed to infections endemic to the areas in which they were operating at the time (*1*). Examples from British military history include many diseases that may be categorized as “undifferentiated febrile illnesses” (*2*). The causative agents for these fevers, often unidentified at the time they occurred, were predominantly infectious pathogens established in the geographic area in which troops were operating. For example, in the late 19th century, the zoonotic disease brucellosis and the vector-borne parasitic diseases leishmaniasis and malaria were common in British colonial troops deployed to the tropics and subtropics. In India, 24% of troops were admitted to the hospital for malaria in 1908 alone (*3*). During World War I, cases of leptospirosis (*4*) and trench nephritis (in retrospect thought to be caused by hantavirus, which resulted in >35,000 illnesses) (*5*) were also documented.

More recently, since World War II, UK military operations in Korea, Malaya, Borneo, Sierra Leone, Liberia, East Timor, and Haiti have resulted in illnesses among military personnel caused by vector-borne pathogens; these illnesses have included Japanese encephalitis, malaria, and dengue (*2*,*6*). This incidence of infectious disease is not limited to the tropics; campaigns in the Balkans and the Arabian Gulf during the past 25 years resulted in reports of cases of a range of vector-borne and zoonotic infections (*7*–*9*).

Although information about endemic infectious disease in Afghanistan is limited, previous historical reports indicate that several endemic vector-borne and zoonotic diseases, including rickettsial diseases, Crimean-Congo hemorrhagic fever (CCHF), and pappataci fever (now known as sandfly fever) (*10*). Given the country’s geographic location, other pathogens endemic to the central Asian region could also be expected to be of concern to military personnel; these pathogens include Sindbis, chikungunya, and West Nile fever viruses (*11*). Although infections with many of these pathogens can be prevented by drugs (chemoprophylaxis) or vaccination, and the risk for exposure can often be reduced by physical measures, the ongoing UK military campaign in Afghanistan has still resulted in infectious diseases occurring in deployed personnel. Severe cases have required restrictions of duties or even hospitalization or evacuation to the United Kingdom, and high illness rates can affect the operational capability of the military force (*12*).

In 2011, Bailey et al. documented numerous cases of “undifferentiated febrile illness” in British military personnel serving in Helmand Province, Afghanistan, during the summer of 2008 (*13*). These cases, for which no organ focus could be determined on clinical and radiologic assessment and no positive results were obtained from microbiological investigations (e.g., blood cultures and malaria antigen tests), have been given the colloquial term “Helmand fever.”

For this study, we conducted surveillance among UK military personnel to determine the prevalence of several vector-borne or zoonotic infectious agents that were suspected to be the causative agents of some of these cases of undifferentiated fever: *Rickettsia* spp., *Coxiella burnetii*, sandfly fever virus, hantavirus, and CCHF virus (CCHFV). We conducted serosurveillance testing of 467 military service members who were deployed to Helmand Province during March 2008–October 2011. In this article, we describe the pathogens for which study participants showed seroconversion before and after a tour of duty and the incidence of those seroconversions within the military population.

## Methods

### Recruitment and Sampling

Study volunteers were recruited from either British Army regiments or Royal Marines units before deployment to Helmand Province, Afghanistan. For each deployment, a research nurse visited the unit and gave study information to the troops. Volunteers were required to give informed consent and to have 1 predeployment blood sample taken. After return from the 6-month tour of duty, volunteers were visited and asked to give a second postdeployment blood sample. At the postdeployment visit, volunteers were also asked to complete a short questionnaire detailing any “flu-like” illness or symptoms they experienced while deployed and any contact they had with livestock, wildlife, or insect vectors. The primary location of each company (i.e., group of ≈100 personnel in which the volunteer was posted) while deployed was also noted. Ethical approval for this survey was provided by the UK National Health Service National Research Ethics Service and Ministry of Defence Research Ethics Committee.

Blood samples were taken by venipuncture using BD SSTII Advance 10-mL vacutainers (Becton Dickinson, Oxford, UK). After a minimum 30-min incubation at room temperature, samples were centrifuged at 3,500 rpm for 15 min to separate the serum. This serum fraction was then separated and stored at −80°C for subsequent serologic testing.

During the study period, March 2008–October 2011, a total of 467 volunteers gave paired serum samples (i.e., predeployment and postdeployment samples). In all cases, samples were kept anonymous (by unique identification numbers), and questionnaire data/confidential information were kept in a secure database with restricted access. 

### Serologic Testing

Postdeployment samples were tested first for antibodies to all pathogens of interest. If results were positive, the corresponding predeployment sample was then tested to ascertain whether the volunteer seroconverted during the tour of duty for which they were recruited to the study or if they were already seropositive (i.e., exposed/seroconverted to that pathogen) before being recruited for the study. A volunteer whose sample was negative before deployment but positive after deployment was deemed to have seroconverted during that particular tour of duty. For each pathogen of interest, diagnostic tests were used in line with Public Health England’s diagnostic laboratory procedures. All assays used have been validated locally for diagnostic use, are approved by UK regulatory authorities, and have been awarded Communauté Européenne marking by the European Union.

### *C. burnetii* ELISA

Antibodies against *C. burnetii*, the causative agent of Q fever, were detected by using a commercial ELISA to detect phase 2 IgG and IgM human antibodies (Serion/Virion, Würzburg, Germany). ELISAs were conducted by using the DS2 Automated ELISA workstation (DYNEX Magellan Biosciences, Chantilly, VA, USA) according to the manufacturer’s instructions. All serum samples were tested at an initial dilution of 1:100, as recommended by the manufacturer’s product insert for diagnostic samples. If results were calculated as positive (above the automated threshold on DS2 software) for either IgG or IgM, the test was repeated in duplicate for confirmation. Because of the nonacute nature of the survey, all samples showing at least Phase 2 IgG positivity were deemed positive.

### Hantavirus Immunofluorescence Assay (IFA)

Antibodies against the hantavirus group of pathogens were detected by using a commercial Hantavirus IIFT Mosaic 1 IgG IFA (Euroimmun, Lübeck, Germany). All serum samples were tested at an initial dilution of 1:100, as recommended by the manufacturer’s product insert for diagnostic samples. If results were calculated as positive (for any species/subspecies of hantavirus), the test was repeated and confirmed by using the Recomline Bunyavirus IgG/IgM Line Assay (Mikrogen, Neuried, Germany). All IFA slides were set up by using the AP16 IF Plus automated slide processor (Euroimmun) and viewed by using a conventional fluorescent light microscope.

### *Rickettsia *IFA

Antibodies against *Rickettsia *spp. were detected by using a commercial Rickettsia IgG and IgM IFA (Focus Diagnostics, Cypress, CA, USA). This assay is specific for rickettsial infection; that is, antibodies against *Coxiella* spp. do not cross-react in this test (Public Health England, unpub. data). All serum samples were tested at an initial dilution of 1:64, as recommended by the manufacturer’s product insert for diagnostic samples. If results were calculated as positive, the test were repeated and confirmed by titrating down a 2-fold dilution series to 1:512 (data not shown) to ensure that fluorescence diminished with antibody dilution (as recommended in the product insert). Because samples were not obtained during an acute illness phase, any sample that showed a consistent signal at 1:64 dilution was considered to be positive, even if this diminished by the 1:128 dilution. All IFA slides were prepared in accordance with the manufacturer’s instructions and viewed by using a conventional fluorescent light microscope

### Sandfly Fever Virus IFA

Antibodies against sandfly fever viruses (genus *Phlebovirus*) were detected in serum by using a commercial sandfly fever assay (IIFT Mosaic 1 IgG immunofluorescence assay; Euroimmun). All serum samples were tested at a dilution of 1:100, as recommended by the manufacturer’s product insert for diagnostic samples. Given the nonacute nature of the samples, a positive result was defined as any sample that showed a consistent signal (for any of the 4 species/strains of sandfly fever virus) at 1:100 dilution. All IFA slides were set up by using the AP16 IF Plus automated slide processor (Euroimmun) and viewed by using a conventional fluorescent light microscope.

### Crimean-Congo Hemorrhagic Fever ELISA

Antibodies against CCHFV nucleoprotein were detected by using an in-house CCHF recombinant ELISA (*14*). ELISAs were carried out by using the DS2 Automated ELISA workstation (DYNEX Magellan Biosciences). All serum samples were tested at an initial dilution of 1:100, and any tests that gave any signal above background were repeated in duplicate.

## Results

The Table shows the results of the respective serology assays performed. For each pathogen (with the exception of CCHFV, for which no seroprevalence was reported), 2 categories of a seropositive volunteer were identified: those who showed detectable levels of antibody before and after deployment; and those who only showed positivity on return, with no detectable antibody in predeployment samples. During deployment, the troops showed the highest seroconversion rates for sandfly fever virus (3.1%) and rickettsiae (2.7%). However, seroconversions for hantavirus and *C. burnetii* (1.3% and 1.7%, respectively) also occurred.

**Table:** Results of antibody testing for 5 infectious pathogens among UK service personnel before and after deployment to Helmand Province, Afghanistan, March 2008–October 2011*

Pathogen	No. persons tested	No. (%) with detectable antibody before deployment	No. (%) with seroconversion after deployment	Total no. (%) with positive antibody test
CCHFV	466	0	0	0
Sandfly fever virus	459	8 (1.7)	14 (3.1)	22 (4.8)
*Rickettsia* spp.	446	10 (2.2)	12 (2.7)	22 (4.9)
Hantavirus	453	5 (1.1)	6 (1.3)	11 (2.4)
*Coxiella burnetii*	467	7 (1.5)	8 (1.7)	15 (3.2)

*Assays were run sequentially on samples from all persons tested; some sample sizes were insufficient for testing for all agents. CCHFV, Crimean-Congo hemorrhagic fever virus.

The group of volunteers who were positive for each pathogen before deployment showed that there was a background level of seroprevalence for these pathogens within the UK military. The group of initially antibody-negative personnel seroconverted to the pathogens while on deployment, which suggests that military personnel are being exposed to such diseases while on operations in Afghanistan and that these diseases are being transmitted in the region. 

**Figure 1:**
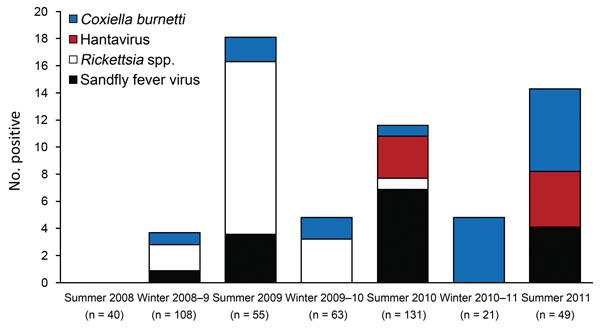
Results of antibody testing for 4 infectious pathogens, by tour of duty, among 467 UK service personnel deployed to Helmand Province, Afghanistan, March 2008–October 2011. n values indicate number of volunteers tested from each tour of duty. Assays were run sequentially on samples from all persons tested; some sample sizes were insufficient for testing for all agents.

Figure 1 shows the prevalence of volunteers who seroconverted during each deployment period. This comparison enabled us to plot prevalence as a function of season: winter deployments (October–March) compared with summer deployments (March–October). This analysis showed a higher prevalence of seroconversion in the Northern Hemisphere summer months, when the deployment area is hotter and drier than over the winter. Sandfly fever virus showed the greatest seasonal variation, with most cases in the summer months, and *C. burnetii* showed the least variations, findings that are consistent with these pathogens’ known modes of transmission.

**Figure 2:**
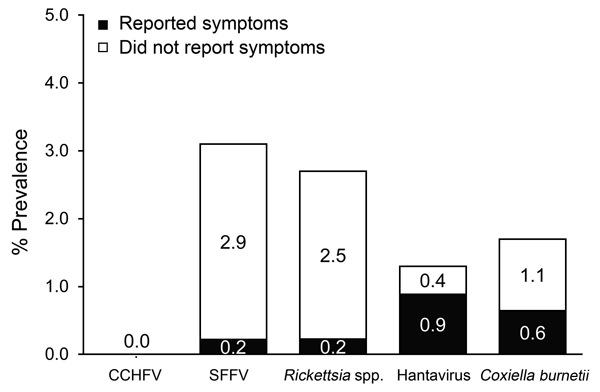
Percentages of UK service personnel who seroconverted to 1 of 5 infectious pathogens who reported feeling unwell or did not report illness during deployment to Helmand Province, Afghanistan, March 2008–October 2011. A total of 90 (19.3%) of 467 deployed service members reported feeling unwell during deployment. CCHFV, Crimean-Congo hemorrhagic fever virus; SFFV, sandfly fever virus.

Many seroconversions for the studied pathogens appeared to be asymptomatic. All volunteers took part in a return questionnaire when the postdeployment sample was taken. This questionnaire was used to determine whether those who seroconverted after deployment experienced a “flu-like” illness during deployment. Figure 2 shows that, for all 4 pathogens acquired (*Rickettsia *spp., *C. burnetii*, sandfly fever virus, and hantavirus), a proportion of volunteers showed seroconversion but did not report feeling ill. As expected, sandfly fever virus and rickettsial infections showed the highest proportion of asymptomatic cases; just 6.5% and 7.4%, respectively, of those who seroconverted reporting feeling ill (i.e., 93.5% of sandfly fever virus exposures and 92.6% of *Rickettsia* spp. exposures were asymptomatic). The less common but often more clinically significant pathogens hantavirus and *C. burnetii* resulted in more reports of illness; 64.7% of *C. burnetii* seroconversions and 30.8% of hantavirus seroconversions were asymptomatic.

**Figure 3:**
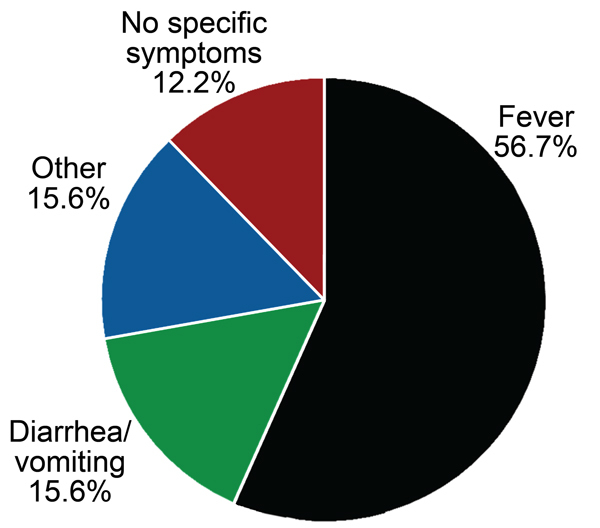
Distribution of signs and symptoms among 90 UK service personnel who seroconverted to 1 of 4 infectious pathogens (sandfly fever virus, hantavirus, *Coxiella burnetii*, *Rickettsia* spp.) and who reported feeling ill during deployment to Helmand Province, Afghanistan, March 2008–October 2011.

Postdeployment questionnaires showed that nearly one fifth (90/467, 19.3%) of all volunteers reported experiencing “flu-like” symptoms while on operations in Helmand Province. A total of 56.7% of those who recorded >1 episodes of feeling unwell reported experiencing fever only, whereas 12.2% had no additional/specific symptoms; the remaining 31.2% reported either diarrhea and vomiting (15.6%) or “other” symptoms (Figure 3).

## Discussion

UK military personnel consist of ≈237,190 persons (estimate of combined regular and volunteer reserve military personnel in UK, 2010 [UK Ministry of Defence, unpub. data]). Our results show that military personnel are being exposed to, at minimum, *Rickettsia* spp., *C. burnetii*, sandfly fever virus, and hantaviruses while on operations in Afghanistan, a region where these agents and resulting diseases are endemic. These results suggest that human or animal (for the zoonotic pathogens) reservoirs likely exist in the local populations of the Helmand region for these diseases.

Troops often patrol through vegetation and farm land where both reservoir and vector populations are high, which can easily result in exposure to pathogens and further supports the hypothesis that the nature of their work makes armed forces personnel a group with an increased incidence of exposure to such vector-borne or zoonotic pathogens. However, this group may also act as a good sentinel cohort for other populations of nonindigenous workers from governmental and nongovernmental organizations. 

UK troops do sometimes stop over at British military bases in Cyprus during their return to the United Kingdom. However, these stopovers are typically very short in duration (2–3 days), and they do not occur in an environment where exposure is likely to occur, with the possible exception of sandfly bites. In addition, for each pathogen studied, a proportion of troops showed antibody positivity before deployment, suggesting that they may have been exposed to these pathogens on previous deployments to Afghanistan (before being recruited to the study) or on other exercises and operations around the world.

Overall, seroprevalence for these pathogens appears to be low, although this is hard to determine without a comprehensive study of nondeploying troops. These results may reflect good discipline or education of UK troops in the use of personal protection measures such as the use of DEET-containing insect repellent and mosquito nets, both of which are actively promoted by the UK military. A higher proportion of those deployed over the summer months showed seroconversion, corresponding with an increase in the numbers of biting vectors such as the *Phlebotomus* sandfly and ticks (rickettsial vectors). Further study of seasonality might show whether these increases correlate with increased military activity (e.g., military patrolling and combat operations increase during the summer months), environmental/entomologic changes (e.g., decreases in rain and/or increases in temperature conditions favorable to agent, reservoir, or host density), or possible lapses in discipline regarding personal protective measures. 

At first glance, summer seasonality may seem odd for an increase in hantavirus seroconversion, for which incidence would be more likely to correlate with closer proximity of humans with the rodent reservoir. However, UK summer deployments extend to late September and in some cases the first week or two of October; thus, these deployment continue into early autumn, when rodents are actively moving into buildings. In addition, the apparent lack of rickettsial infections after summer 2010 raises questions of whether this might correlate with a change in vector life-cycle, abundance of reservoir species, changes in insect bite prevention regimens, or changes to local livestock management regimens.

The increased incidence of rickettsial and sandfly fever virus infections that we found among deployed troops does not necessarily mean that these vector-borne pathogens are more prevalent in the environment and local population than the zoonotic pathogens *C. burnetii* or hantavirus. Rather, these results may mean that mode of transmission and incidence of encounters with the vectors and reservoirs differ. However, our results do indicate that these pathogens may pose the greatest risk to UK troops.

Although most of the diseases we detected are relatively self-limiting after the initial acute infection, our results suggest ways to improve control measures to reduce the rate of transmission. UK military personnel are already given medical advice and education before deployment, emphasizing the prevention of insect bites while deployed by use of arthropod repellent, insecticide-impregnated clothing, and mosquito nets. The observation that rickettsial infections and Q fever might account for a sizeable proportion of cases of undifferentiated febrile illness seen in military field hospitals has led to the empirical use of doxycycline in such cases because *Rickettsia* and *Coxiella* species are sensitive to this drug. This practice justifies further research on use of doxycycline for chemoprophylaxis in these instances.

More than half (56.7%) of volunteers who reported feeling unwell specifically reported a fever without other specific symptoms, which is consistent with the “undifferentiated febrile illness” that the pathogens we investigated can cause. The 15.6% of volunteers reporting illness who reported diarrhea and vomiting could have had gastrointestinal infections; localized outbreaks of norovirus and similar infections are common in military operating bases) (*15*). Of the many volunteers who experienced fever but did not show seroconversion for the 5 pathogens tested here, exposure to influenza virus or other respiratory infections should be considered. In 2011, Eick et al. reported that deployed US troops experienced 30.1% seroconversion for influenza and 6 other respiratory infections (*16*). However, uptake of influenza vaccination, which is offered to UK military personnel before winter deployments, has increased for each of the past 4 years (*2*). Data from the militaries of other countries, which might have different deployment patterns and protective measures, should be compared only with caution. In addition, although some volunteers who seroconverted for all 4 pathogens reported flu-like symptoms while deployed, we cannot ascertain whether these symptoms were a result of exposure to the pathogen of interest.

Some volunteers who seroconverted for the pathogens tested were asymptomatic for the duration of their deployments. Clinical disease probably did not develop in these patients, despite evidence of an exposure and an antibody response. Our finding that 65% of acute *C. burnetii* infections were asymptomatic is consistent with previous reports (*17*,*18*), but even in asymptomatic persons infected with this pathogen, long-term complications such as chronic Q fever and Q fever fatigue syndrome may develop. Overall, ≈5% of acute cases progress to chronic Q fever, and complications can include endocarditis requiring prolonged antimicrobial drug treatment and possibly heart valve surgery (*19*). In addition, Q fever fatigue syndrome may develop in ≈20% of those infected (*17*), which is generally incompatible with a military career and has substantial effects on patients’ quality of life. 

Royal et al. (*20*) recently reported *C. burnetii* seroprevalence in US troops deployed to the Al Asad region of Iraq in 2005; a known Q fever outbreak occurred in this region at this time. This smaller study (n = 136) reported a 7.2% prevalence of *C. burnetii* infection among troops located in that area at the time of the outbreak. The report further supports (in addition to original work by Bailey et al. [*13*]) the *C. burnetii* seroprevalence reported here. Although we did not see as high an incidence of seroconversion, we were not specifically looking in an area with a known Q fever outbreak, merely an area in which Q fever is believed to be endemic.

Although this study did not find seroconversion for the viral hemorrhagic fever agent CCHFV, military and public health reports demonstrated that the virus is circulating in the region (*21*). In 2009, a US Army soldier in the neighboring Kandahar Province died of CCHFV infection (*22*), and in 2012, a UK citizen returning from the northwest of the country also died (*23*,*24*).

In conclusion, this study highlights and confirms the potential for vector-borne and zoonotic diseases that are endemic in southern Afghanistan to emerge or reemerge to pose a substantial public health threats as the country rebuilds its public health infrastructure. A study of this type cannot give a specific indication of prevalence for these pathogens in the local population, but this surveillance can provide a valuable way of exploring emerging disease epidemiology, particularly of vector-borne and zoonotic infections, in areas with poor public health reporting and infrastructure. Our findings of seroconversion for 4 of these pathogens among deployed UK troops reinforce the need for continued surveillance and continued education of health care providers so that, should military operations or environmental factors change in such a way that these modest incidence numbers increase, costly outbreaks can be avoided. This study also highlights the need for rapid, field-capable, point-of-care diagnostics in regions or situations for which full laboratory diagnostic facilities are not practical or available. 
